# Arthroscopic decompression of an entrapped suprascapular nerve due to an ossified superior transverse scapular ligament: a case report

**DOI:** 10.4076/1757-1626-2-8200

**Published:** 2009-08-06

**Authors:** Neoptolemos N Sergides, Dimitrios D Nikolopoulos, Euangelos Boukoros, George Papagiannopoulos

**Affiliations:** Department of Orthopaedic, Shoulder and Knee Arthroscopy Division, Central Clinic of AthensAsklipiou, 31 Str, 106-80Greece

## Abstract

**Introduction:**

Suprascapular neuropathy is an uncommon cause of shoulder pain and weakness and therefore is frequently misdiagnosed. As a consequence, misdiagnosis can include inappropriate conservative treatment or unsuccessful surgical procedure.

**Case presentation:**

A rare case is reported of a 54-year-old woman who suffered from suprascapular nerve entrapment syndrome. The patient was subjected to arthroscopy of the left shoulder, where a compression of the suprascapular nerve due to an ossified superior transverse scapular ligament was diagnosed. The arthroscopic release of the suprascapular nerve brought relief from pain, weakness and atrophy of the supraspinatus and infraspinatus muscles.

**Conclusion:**

Arthroscopic decompression of the entrapped suprascapular nerve is technically challenging, but less invasive and potentially a more effective way to treat suprascapular neuropathy, as it may provide a more rapid recovery, especially in the rare case that the nerve is depressed by an ossified superior transverse scapular ligament.

## Introduction

Suprascapular nerve (SSN) entrapment was firstly reported in 1959, by Koppel and Thompson [1]. Suprascapular nerve entrapment syndrome (SNES) can be caused by a variety of anatomic and pathologic entities as the nerve courses from the brachial plexus (C5-C6) through the suprascapular and spinoglenoid notches to innervate the supraspinatus and infraspinatus muscles. Suprascapular neuropathy can be presented as pain located in the posterior and lateral aspect of the shoulder; as weakness, with little or no pain; whereas other patients may be completely asymptomatic [2-4]. In the present study, ossification of the superior transverse scapular ligament (STSL) is the cause of the SNES. There has been reported some cases with ossification of STSL [5-8], but this is the first study for arthroscopic decompression of a patient suffering from SNES due to an ossified STSL.

## Case presentation

A 54-year-old female Caucasian patient presented to the orthopaedic department of the clinic complaining of a six month left shoulder pain, especially localized in the scapular region, and a progressive weakness of the left upper extremity. She recalled no trauma to the shoulder and the symptoms were aggravated by lifting objects overhead. Physical examination revealed full passive ROM, but significant weakness was found in abduction (60°) and in external rotation (15°). Moderate atrophy of the supraspinatus and infraspinatus was also found. Manuel test strength resulted 3/5 for supraspinatus and infraspinatus. There was no sensory loss detectable in the left upper extremity, nor reflex changes.

X-rays of the cervical spine and shoulder region were normal. Electromyography revealed denervative changes with fibrillation and P waves on rest and reduction of motor units on active movement of the supraspinatus and infraspinatus muscles. Nerve conduction studies showed slowing of SSN motor conduction [11.5 (normal value from 3.3 ± 0.5)]. An MRI of the left shoulder revealed moderate rotator cuff tear and changes in the supraspinatus and infraspinatus muscles secondary to atrophy (denervation). These changes include decreased muscle bulk, fatty infiltration, and homogeneous high signal intensity on T2-weighted images. MRI did not identify any specific pathology of the suprascapular notch area (tumors or ganglion cysts, scapular bone’s congenital structural changes), nor the STSL (thickening or ossification).

**Figure 1.**
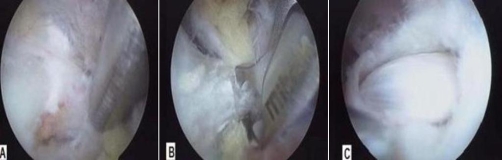
An entrapped SSN due to STSL ossification. The suprascapular artery passes over the STSL **(A)**. The STSL was sectioned using an arthroscopic scissor and a 4 mm spine punch **(B)**. The uncompressed SSN, after scissoring the STSL **(C)**.

The patient was arthroscopically treated. Five portals were used: (i) the classic posterior portal (typically used to visualize the glenohumeral joint and the subacromial space), (ii) a lateral subacromial portal, (iii) an anterior portal (iv) the Neviaser portal and (v) the SSN portal described by Lafosse and Tomasi [9]. After inspection of the glenohumeral joint, the arthroscope was introduced into the subacromial space till the suprascapular notch. The STSL was found ossified and the SSN was entrapped as the amount of space around it was extremely limited (Figure 1a). The ligament was sectioned using an arthroscopic scissor and a 4 mm spine punch (Figure 1b,1c). Finally, the rotator cuff rupture was repaired. The patient had immediate pain relief after surgery (VAS <4) and was discharged on the following day. On the 6^th^ month an EMG was obtained and revealed significant improvement of the SSN. The patient returned to her daily activities 3 months after surgery.

## Discussion

The SSN is a mixed motor and sensory peripheral nerve arising from the superior trunk (fifth and sixth cervical nerves) of the brachial plexus (Erb’s point) with a variable contribution from the fourth cervical nerve root [2,3]. The nerve travels below the transverse scapular ligament as it crosses the suprascapular notch to enter the supraspinatus fossa, whereas the suprascapular artery usually travels above the ligament [3,4]. The classic description of the STSL is completely non-ossified single band and should be expected, on average, in three fourths of the cases [2,3]. Partial or complete ossification (as in our case study) and anomalous bifid or trifid bands of the STSL have been described [5-8]. The nerve then traverses the supraspinatus fossa, giving motor branches to the supraspinatus, with variable minor sensory contributions to the glenohumeral and acromioclavicular joints, and occasionally to the skin [2,3]. The nerve angles around the spine of the scapula at the spinoglenoid notch, traveling with the artery under the spinoglenoid ligament. The motor branches to the supraspinatus are approximately 1 cm from the suprascapular notch, 3 cm from the origin of the long head of the biceps, whereas the motor branches to the infraspinatus average 2 cm from the posterior glenoid rim [2-4,8].

Peripheral nerves are highly susceptible to injury from stretch and compression. Both of these mechanisms result in nerve ischemia, edema, micro-environmental changes, and conduction impairment [2]. Pressure on the SSN has been reported to be due to: (i) congenital structural changes of the scapular bone, (ii) fracture of the scapula that produces modification of the anatomic structure of the notch, (iii) tumors or ganglion cysts usually at the suprascapular or spinoglenoid notch (the most commonly reported mechanism of injury), (iv) the sling effect phenomenon produced by SSN being fixed at two points, one in the brachial plexus and the second in the supraspinatus muscle, preventing the nerve from moving in the notch. Vascular micro-trauma (v) has also been postulated to cause nerve dysfunction. A thickened or calcified suprascapular ligament (vi) may also compress the nerve. There may also be dynamic compression from the spinoglenoid ligament as proposed recently [2-4,8].

Diagnosing SNES can be very difficult. A detailed history and physical examination are required. Radiographs, MRI, electromyography and nerve conduction studies can provide essential information in the diagnosis of the SNES [2-4]. Suprascapular neuropathy frequently presents as a poorly localized dull ache over the lateral and posterior aspects of the shoulder associated with weakness of external rotation and abduction, which can mimic rotator cuff tears or cervical disc disease. The pain and weakness are more severe with pathologic changes at the level of the suprascapular notch than at the spinoglenoid notch. Symptoms can be referred to the lateral aspect of the arm, the ipsilateral side of the neck, or the anterior chest wall. Some patients present with painless isolated wasting of the infraspinatus [2-4,8]. A history of trauma -a direct impact on the shoulder or from indirect force, such as a fall on an outstretched arm- or repetitive use of the shoulder -including volleyball, handball, tennis, weight lifting, swimming, heavy labor- is common [2-4,8].

The initial treatment for suprascapular neuropathy without evidence of a space-occupying lesion should be nonoperative, with activity modification, anti-inflammatory and analgesic medications, combined with physiotherapy [2,3,9]. Most suprascapular neuropathies resolve completely. Unfortunately, neuropathic symptoms, including pain and weakness, may take more than a year to reach maximum improvement. A self-directed home exercise program of physical therapy is prescribed to maintain full glenohumeral motion and to strengthen the rotator cuff muscles, the deltoid, and the periscapular musculature [2,3]. Special attention should be directed toward establishing proper posture with scapular retraction exercises, as well as strengthening of the trapezius, the rhomboids, and the serratus musculature. Rehabilitation focused on scapular function is beneficial in recovery and may avoid recurrence of the injury [2,4].

Surgical intervention is indicated when there is no improvement after 6 months of nonsurgical care or in cases with clear evidence of a compressive lesion. Decompression of the SSN at the suprascapular notch can be achieved even by an open surgical intervention through trapezius-splitting approach or by arthroscopic dissection of the STSL [9,10]. Care must be taken to avoid injury to the suprascapular artery and vein that travel superficial to the transverse scapular ligament.

## Conclusion

Suprascapular neuropathy should be considered in the differential diagnosis of shoulder pain, especially when other common causes of shoulder pain have been excluded. The SNES could be due to compression, traction, or injury from direct trauma. An ossified STSL can be another cause of suprascapular neuropathy, although rare.

During routine shoulder arthroscopy deliberate attempts to explore SSN should not be made; notwithstanding every time that a suprascapular neuropathy is suspected, then it is very helpful to explore arthroscopically the SSN, and if any pathology is found to be repaired.

It is sure that arthroscopic decompression of the SSN is technically challenging, but less invasive and potentially a more effective way to treat suprascapular neuropathy, as it may provide a more rapid recovery, especially in the rare case that the nerve is entrapped by an ossified STSL.

## Abbreviations

EMG: electromyography
MRI: magnetic resonance imaging
ROM: range of motion
SNES: Suprascapular nerve entrapment syndrome
SSN: suprascapular nerve
STSL: superior transverse scapular ligament
VAS: visual analog scale
